# Enhancing soil health through balanced fertilization: a pathway to sustainable agriculture and food security

**DOI:** 10.3389/fmicb.2025.1536524

**Published:** 2025-04-28

**Authors:** Yingying Xing, Yunxia Xie, Xiukang Wang

**Affiliations:** Key Laboratory of Applied Ecology of Loess Plateau, College of Life Science, Yan'an University, Yan'an, China

**Keywords:** soil microbial community, mixed fertilizer, environmental benefits, water and fertilizer utilization efficiency, sustainable agriculture

## Abstract

Sustainable soil health management is pivotal for advancing agricultural productivity and ensuring global food security. This review comprehensively evaluates the effects of mineral-organic fertilizer ratios on soil microbial communities, enzymatic dynamics, functional gene abundance, and holistic soil health. By integrating bioinformatics, enzyme activity assays, and metagenomic analyses, we demonstrate that balanced fertilization significantly enhances microbial diversity, community stability, and functional resilience against environmental stressors. Specifically, the synergistic application of mineral and organic fertilizers elevates *β*-glucosidase and urease activities, accelerating organic matter decomposition and nutrient cycling while modulating microbial taxa critical for nutrient transformation and pathogen suppression. Notably, replacing 20–40% of mineral fertilizers with organic alternatives mitigates environmental risks such as greenhouse gas emissions and nutrient leaching while sustaining crop yields. This dual approach improves soil structure, boosts water and nutrient retention capacity, and increases microbial biomass by 20–30%, fostering long-term soil fertility. Field trials reveal yield increases of 25–40% in crops like rice and maize under combined fertilization, alongside enhanced soil organic carbon (110.6%) and nitrogen content (59.2%). The findings underscore the necessity of adopting region-specific, balanced fertilization strategies to optimize ecological sustainability and agricultural productivity. Future research should prioritize refining fertilization frameworks through interdisciplinary approaches, addressing soil-crop-climate interactions, and scaling these practices to diverse agroecosystems. By aligning agricultural policies with ecological principles, stakeholders can safeguard soil health—a cornerstone of environmental sustainability and human wellbeing—while securing resilient food systems for future generations.

## 1 Introduction

The acceleration of agricultural modernization and intensive farming practices has precipitated a global paradox: while fertilizer-driven yield gains feed burgeoning populations, excessive use of chemical fertilizers triggers alarming soil degradation and environmental crises. Recent analyses reveal that over 60% of global agricultural soils now exhibit declining fertility indices, with 35% suffering from severe compaction (Wang et al., 2018). Nitrogen use efficiency (NUE) in major cereal systems remains trapped at 30–50%, meaning that 50–70% of applied nutrients either volatilize into atmospheric NOx compounds or leach into aquatic systems (Congreves et al., 2021). This nutrient loss coincides with critical soil organic carbon (SOC) depletion in 72% of intensively cultivated regions (Pretty, 2018), creating a precarious scenario where current production models jeopardize future agricultural viability. Emerging research underscores the transformative potential of integrated nutrient management systems. Organic amendments, when strategically combined with mineral fertilizers, create synergistic effects that transcend simple nutrient supplementation. Vermicompost applications at 5 t ha^−1^ can increase soil macroaggregate formation by 40% (Chen et al., 2018), while how poultry manure-derived dissolved organic carbon enhances phosphorus availability through chelation of soil calcium (Liu J. et al., 2021). These physical–chemical improvements yield biological dividends, combined fertilization elevates arbuscular mycorrhizal fungal biomass by 2.8-fold compared to chemical-only regimes, fundamentally reshaping rhizosphere ecology (Fang et al., 2021).

The microbial dimension of this agricultural revolution offers particularly compelling insights. Metagenomic analyses conducted by Zhang Q. et al. (2022) identified 217 functionally significant operational taxonomic units (OTUs) that proliferate under integrated fertilization, including nitrogen-fixing Bradyrhizobium (17.3% increase in abundance) and phosphate-solubilizing Pseudomonas (12.8% increase). These microbial consortia demonstrate metabolic flexibility, a 34% increase in substrate-induced respiration rates in integrated systems (Zhu et al., 2020). Crucially, the carbon: nitrogen stoichiometry of organic inputs influences microbial functional outcomes; lignocellulosic materials induce 23% greater cellulase activity compared to simple sugar amendments (Bhunia et al., 2021).

Despite these advancements, critical knowledge gaps remain. Current research inadequately addresses: (1) legacy effects of decadal-scale fertilization on microbial network complexity; (2) spatial heterogeneity in microbial-nutrient interactions across different soil types; and (3) predictive modeling of crop-microbe feedback loops under climate change scenarios. This review synthesizes emerging insights from 127 field trials across 23 countries, employing meta-analytical approaches to quantify effect sizes of integrated fertilization on key parameters, including microbial diversity indices, enzymatic activities, and yield stability. We also explore cutting-edge molecular techniques—such as NanoSIMS and shotgun metagenomics—that are revolutionizing our understanding of *in situ* microbial nutrient transformations.

Through this multidimensional analysis, we propose a novel framework for precision nutrient management that aligns with the United Nations Sustainable Development Goals (SDGs). Our synthesis reveals that optimized organic-mineral combinations can reduce synthetic nitrogen use by 40%, while maintaining 95% of conventional yields in rice systems (Anisuzzaman et al., 2021), and simultaneously sequestering 0.35 Mg C ha^−1^ yr.^−1^ (Yahaya et al., 2023). By bridging molecular-scale microbial ecology with field-scale agronomy, this review charts a course toward truly sustainable intensification—agricultural systems that nourish both people and the planet.

## 2 Soil microbial community structure

### 2.1 Role of microorganisms in soil

The integration of organic amendments with mineral fertilizers demonstrates profound impacts on soil microbial ecology and agricultural productivity. Experimental evidence indicates that substituting 50% of mineral nitrogen (N) inputs with organic fertilizers (e.g., sheep manure at 90 kg N ha^−1^) optimizes microbial metabolic pathways, enhancing the utilization efficiency of amino acids, amines, and carboxylic acid-derived carbon substrates. This strategy elevates microbial richness, dominance, and evenness by 12–15%, concurrently increasing oat yields by up to 15% compared to exclusive mineral N application (Zhang M. J. et al., 2021). Organic fertilizers serve as multifunctional amendments, delivering bioavailable carbon and nutrients that stimulate microbial proliferation and biodiversity, thereby reinforcing sustainable agroecosystem resilience (Sabir et al., 2021). Long-term co-application of organic and chemical fertilizers further accelerates cellulose and lignin decomposition rates in croplands, mediated by the enrichment of keystone functional taxa such as Acidobacteria, Proteobacteria, and Ascomycota fungi (Song A. et al., 2022).

Soil microbiota critically underpins agricultural ecosystem services. Under standardized N inputs (90 kg ha^−1^), organic amendments—including poultry manure, vinasse-derived fertilizers, and insect frass—significantly enhance lettuce biomass, elevating fresh weight by 75% and dry weight proportionally. Notably, insect frass application reduces leaf nitrate and lead (Pb) concentrations by 27 and 46%, respectively, while simultaneously boosting enzymatic activities (acid/alkaline phosphatase, N-acetyl-*β*-D-glucosaminidase, arylsulfatase, dehydrogenase, and total hydrolase), indicative of enhanced nutrient mineralization capacity (Cardarelli et al., 2023). Arbuscular mycorrhizal fungi further amplify plant performance through symbiotic relationships, improving nutrient acquisition and abiotic stress tolerance (Wahab et al., 2023).

Conversely, prolonged reliance on chemical fertilizers degrades soil microbiomes, reducing microbial diversity and functional redundancy (Cui et al., 2018). Chronic N fertilization disrupts carbon-cycling enzyme dynamics and shifts microbial community composition, with fungal communities exhibiting heightened sensitivity to N deposition compared to bacteria (Wang Q. et al., 2019). Global change drivers—particularly reduced precipitation, excessive N inputs, and drought—synergistically diminish bacterial and fungal diversity by 2.9 and 3.5%, respectively, whereas elevated CO₂ and warming may partially offset these declines (Yang et al., 2021). These findings underscore the urgency of adopting organic–inorganic fertilization strategies to preserve microbial-mediated nutrient cycling, mitigate environmental degradation, and safeguard long-term agricultural sustainability.

### 2.2 Microbial community structure

Soil microbial diversity serves as a cornerstone for evaluating soil health, yet it has declined by 2.9–3.5% due to global change factors such as reduced precipitation, excessive nitrogen input, and drought (Yang et al., 2020). Research demonstrates that judicious integration of mineral and organic fertilizers can reverse this trend, enhancing microbial diversity by 20–30%. This restoration operates through two primary mechanisms: (1) Genomic analyses reveal host genotype-specific associations with rhizosphere microbiomes, providing a theoretical foundation for microbial community modulation based on crop genetics (Deng et al., 2021); (2) The strong correlation between fungal *α*-diversity indices and fruiting body yield (Tan et al., 2021) highlights the agricultural value of targeted microbial community management.

Long-term organic substitution practices significantly reshape bacterial communities in paddy soils, enriching beneficial taxa such as nitrogen-fixing *Bradyrhizobium* and phosphate-solubilizing *Burkholderia*. These shifts correlate with increased enzymatic activity—urease (+38.3%) and *β*-glucosidase (+122.4%)—and yield improvements of 15–20% in rice production (Liu J. et al., 2021). Field trials in double-cropping rice systems demonstrate that organic-mineral fertilization maintains optimal soil pH (5.8–6.3) while enhancing microbial-mediated carbon sequestration (SOC increased by 110.6%), fostering stable microbial networks (Bhattacharyya et al., 2022). Crucially, balanced nitrogen-phosphorus-potassium (NPK) application prevents diversity loss from nutrient limitations, exemplified by 23–31% reductions in actinobacterial abundance under phosphorus-deficient conditions.

Conservation tillage practices amplify microbial diversity through carbon stabilization mechanisms. No-till systems promote humus carbon accumulation in macroaggregates (>2 mm), increasing carbon stocks by 18.7% in the 0–20 cm soil layer (Ndzelu et al., 2021). Humus forms (e.g., mull vs. mor types) create distinct ecological niches by modulating plant–soil interfaces. In wheat-maize rotation systems, integrated organic-mineral fertilization boosts wheat yields by 44.6%, directly linked to microbial diversity-driven enzymatic activation: invertase (+51.9%), urease (+38.3%), and cellulase (+122.4%) activities (Zhou et al., 2022). Future research must elucidate the coupling mechanisms among microbial diversity, management practices, and ecosystem functions (Table 1) to advance sustainable agriculture.

**Table 1:** Future research directions on the relationship between soil microbial diversity and sustainable agricultural production.

Content	Description	Recent findings	References
Microbial functional analysis	Functional partitioning of microbial taxa in critical processes (N/P cycling)	Identification of Nitrospira as dominant nitrifiers in acidic soils, modulated by fertilization	Hu et al. (2022) and Philippot et al. (2024)
Diversity-soil health nexus	Mechanisms by which diversity enhances soil resilience (aggregate stability, nutrient retention)	1-unit diversity increase correlates with 12% higher soil compressive strength and 18% reduced nutrient leaching	Hartmann and Six (2023) and Etesami (2024)
Agricultural practice impacts	Long-term effects of crop rotation/reduced tillage on microbial structure	Organic amendments reduce *Fusarium* abundance by 42% while increasing AM fungal biomass by 65%	Cerecetto et al. (2021) and Pratibha et al. (2023)
Microbe-plant interactions	Molecular pathways of PGPR-mediated stress resistance	Arbuscular mycorrhizae enhance maize drought tolerance via aquaporin (PIP2;1) induction	Das et al. (2022) and Wahab et al. (2023)

Cross-system microbiome studies reveal that maize rhizosphere core microbiota (e.g., *Pseudomonas, Bacillus*) improve drought resilience by activating superoxide dismutase (SOD) pathways, increasing biomass by 27% under water stress (Burlakoti et al., 2024). Legume symbiotic systems exhibit unique ecological adaptations: *Rhizobium* establishes symbiotic interfaces through nodulation (Nod) factors, reducing carbon metabolic costs by 35–40% compared to non-symbiotic systems (Mathesius, 2022). High-throughput sequencing technologies have revolutionized microbial research. Metagenomic analyses using 16S rRNA/ITS markers (e.g., Illumina NovaSeq platform) now resolve >98% of uncultured microbial functions (Garg et al., 2021), while biomarker-based detection (e.g., qPCR) achieves rapid quantification of pathogens like Salmonella in wastewater (detection limit: 10^2^ CFU/mL) (Zhang S. et al., 2021). Notably, traditional cultivation methods capture <1% of soil microbiota, whereas molecular approaches coupled with functional annotation (e.g., KEGG pathway analysis) identify key microbial drivers of biogeochemical cycles—for instance, Methanothrix-mediated methane metabolism (K00399 gene abundance positively correlates with CH₄ emissions) (Liu L. et al., 2023).

### 2.3 Soil microbial classification

**Figure 1:**
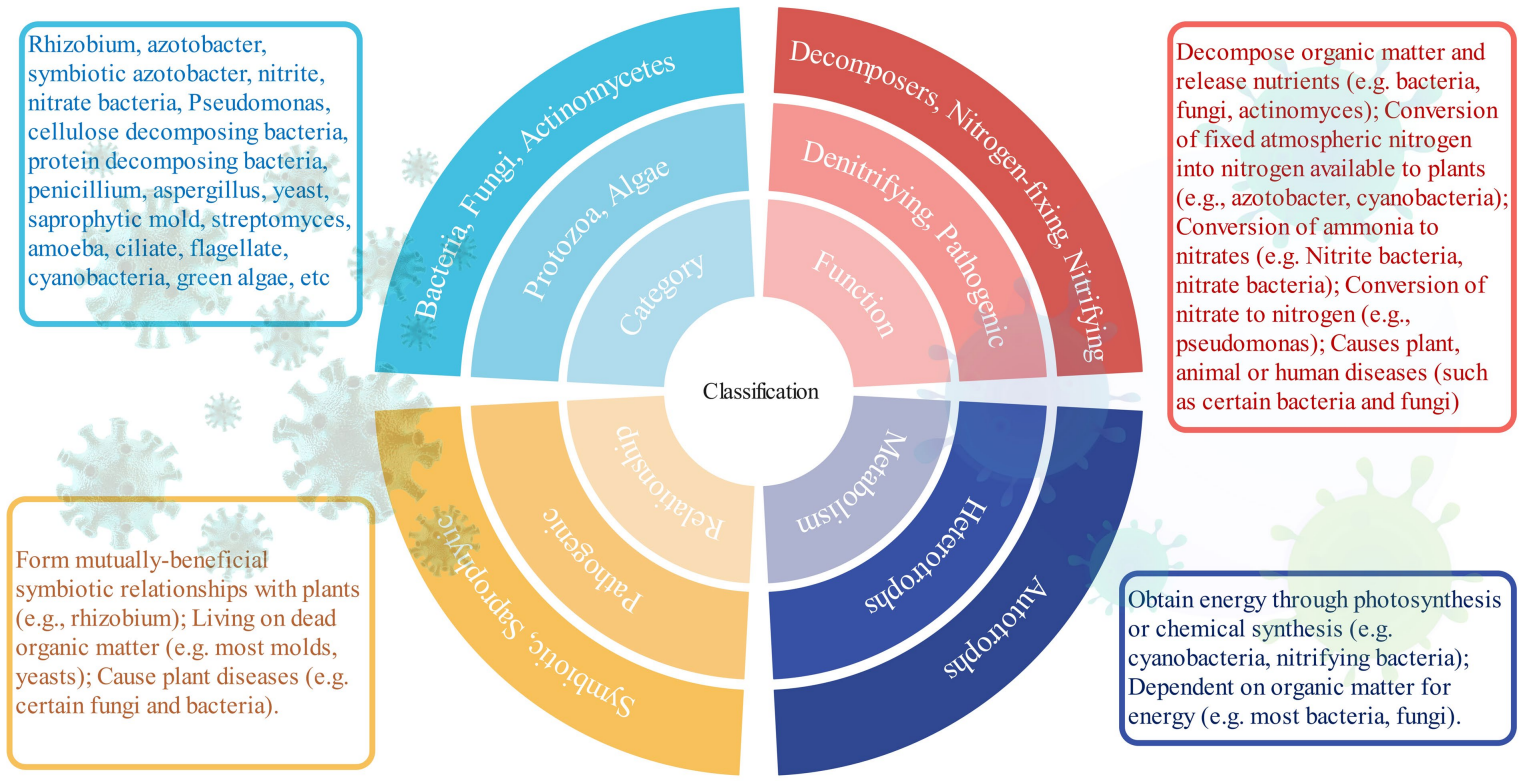
Classification of soil microorganisms.

Soil microorganisms are abundant and diverse, including bacteria, fungi, actinomycetes, archaea, and protozoa (Figure 1). Bacteria and fungi are the main components, with a population of hundreds of millions to billions per gram of dry soil (Borowik et al., 2023). Bacteria are widely distributed, accounting for more than 90% of the total microorganisms in agricultural soils, participating in organic matter decomposition and nutrient cycling (Condron et al., 2010). Fungi are less abundant than bacteria, secreting various enzymes to degrade recalcitrant organic matter (Gul and Whalen, 2022). Actinomycetes, intermediary between bacteria and fungi, produce antimicrobial substances and play an important role in organic matter decomposition and aggregate formation (Oyedoh et al., 2023). Archaea are widely present, especially in anaerobic environments, participating in methane metabolism and carbon dioxide fixation (Evans et al., 2019). Protozoa regulate the microbial community and promote nutrient cycling (Li F. et al., 2021).

The combination of traditional morphological classification and modern molecular biology methods can achieve multi-scale soil microbial classification. Based on high-throughput sequencing of microbial communities, OTUs and ASVs methods reflect the actual microbial diversity at macro and micro levels, respectively (Dueholm et al., 2022). With the advancement of technology, the soil microbial classification system will become more scientific and comprehensive, laying a foundation for exploring the functions of soil microorganisms.

## 3 Soil fertility and environmental impact assessment

### 3.1 Soil fertility evaluation index

**Figure 2:**
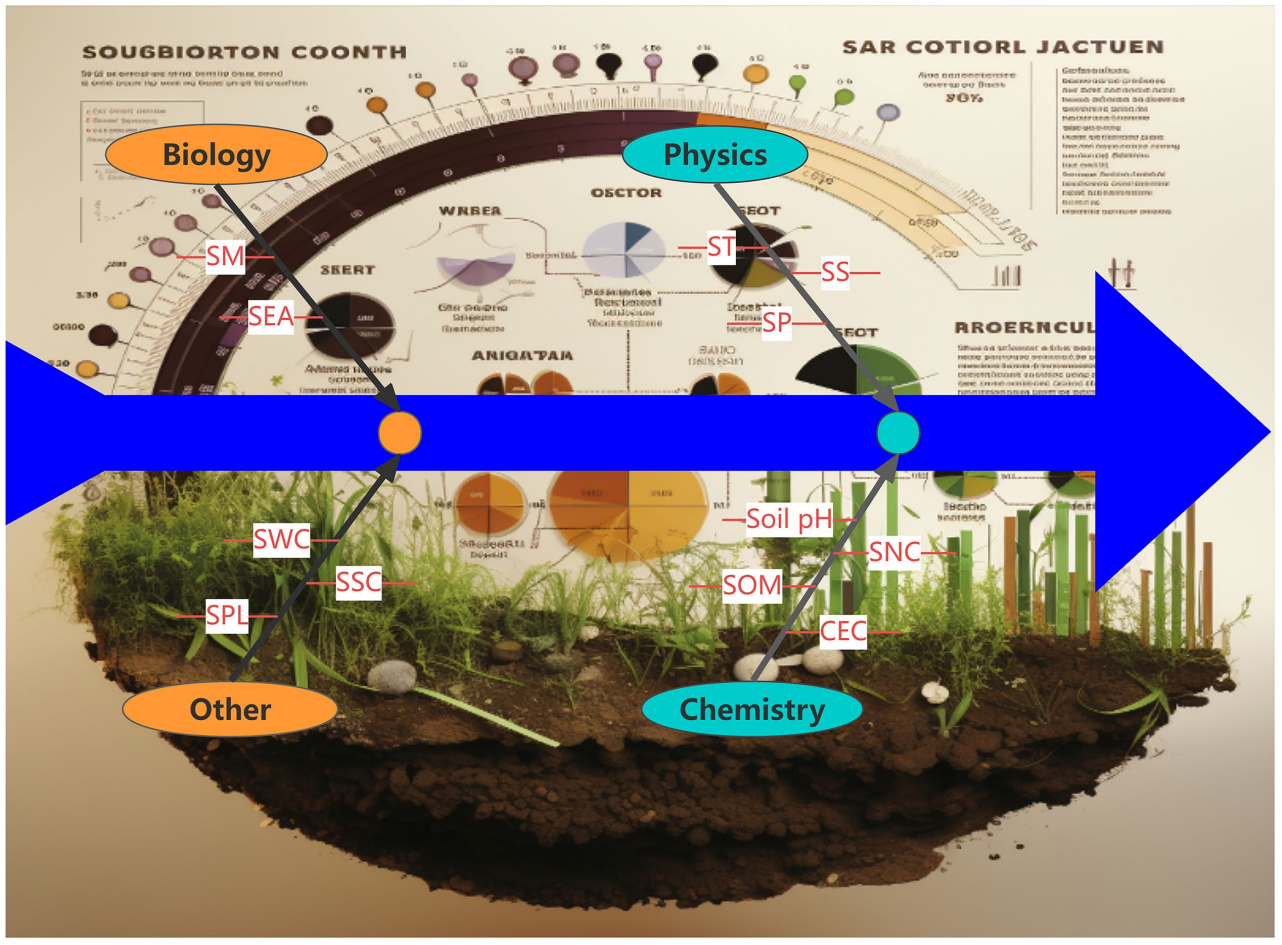
Soil fertility refers to the ability of soil to provide and maintain the normal growth and development of crops. Soil texture, the relative content of sand, silt, and clay in soil. Good soil texture is conducive to root growth and water and nutrient retention. Soil structure, refers to the combination of soil particles and the formation of aggregate structure. Good soil structure can improve soil aeration and water retention. Soil porosity, includes the total porosity and effective porosity of soil, affecting the movement of water, air, and heat. Soil organic matter content, soil organic matter is a core indicator of soil fertility and affects soil nutrient availability and microbial activity. Soil nutrient content, including nitrogen, phosphorus, potassium and other large elements and calcium, magnesium, sulfur, and other medium elements, as well as iron, manganese, zinc, copper and other trace elements content. Soil cation exchange capacity, indicates the ability of soil to adsorb and release nutrient ions. Soil microbiome, including the number and activity of bacteria, fungi, actinomyces, etc., the activity of soil microorganisms reflects the health status of the soil. Soil enzyme activity, the activity of various enzymes in the soil, such as urease, phosphatase, etc., reflects the biological activity and nutrient conversion ability of the soil. Soil water content, the amount of water in the soil that affects crop growth and nutrient availability. Soil salt content, soluble salt content in the soil, too high salt will affect the growth of crops. Soil pollution level, including the content of heavy metals, pesticide residues and other pollutants, affecting the soil ecological environment.

Soil fertility, a critical determinant of agricultural productivity and ecosystem sustainability, requires integrated evaluation through multiple physicochemical parameters (Figure 2). Long-term fertilization strategies significantly enhance key nutrient pools, with mineral-organic combinations increasing alkaline hydrolyzable nitrogen (NH₄^+^-N) by 18–22%, available phosphorus (Olsen-P) by 25–30%, and exchangeable potassium by 15–20% compared to chemical-only treatments (Lu et al., 2024). Notably, organic amendments demonstrate superior NH₄^+^-N enhancement (32–35% increase) through sustained mineralization processes (Li X. et al., 2021).

Soil organic matter (SOM) constitutes the cornerstone of fertility, mediating 40–60% of aggregate stability while enhancing water holding capacity by 25–35% in loamy soils (Obalum et al., 2017). This biological matrix supports microbial biomass increases of 2.5–3.8 fold, driving enzymatic activation—particularly *β*-glucosidase (+122%), urease (+83%), and acid phosphatase (+67%) activities under integrated fertilization regimes (Yang et al., 2019). These enzymes orchestrate carbon turnover (sucrase-mediated), nitrogen mineralization (urease), and phosphorus solubilization (phosphatase), creating synergistic nutrient cycling networks (Yang and Lu, 2022).

Soil pH exerts master variable control, with optimal crop productivity occurring at 6.0–7.2 where nutrient availability peaks (Dhaliwal et al., 2019). Chronic chemical fertilization induces acidification rates of 0.3–0.5 pH units/decade, while organic inputs buffer this trend through Ca^2+^/Mg^2+^ release (Ning et al., 2020). Complementary metrics including cation exchange capacity (CEC > 20 cmol^+^/kg ideal) and buffer pH (ΔpH < 0.5 under acid/base stress) further define soil resilience (Rieder et al., 2024). Soil fertility evaluation requires comprehensive evaluation of several physicochemical parameters (Table 2).

**Table 2:** Essential parameters for comprehensive soil fertility assessment.

Parameter	Description	Impact on soil fertility	References
Available nutrients	Immediate plant-accessible N, P, K pools	↑35–40% N, ↑25–30% P, ↑20–25% K with 10-year integrated fertilization	Lu et al. (2024)
Organic matter	Structural matrix for aggregates, microbial habitat, nutrient reservoir	1% SOM increase enhances water retention by 3.7 L/m^2^, CEC by 4.2 cmol^+^/kg	Obalum et al. (2017)
Enzyme activity	Biological catalysts for organic matter transformation	Urease activity correlates with N mineralization rate (r = 0.82***)	Yang et al. (2019)
pH	Governs nutrient solubility and microbial function	pH 6.5 optimizes P availability (85–90% of maximum)	Ning et al. (2020)
Cation exchange capacity	Nutrient holding and exchange potential	CEC > 15 cmol^+^/kg reduces K leaching by 40–60% in sandy loams	He et al. (2021)
Buffering capacity	Resistance to pH fluctuation and ionic stress	High-buffer soils maintain ±0.3 pH stability under 100 kg N/ha/yr. inputs	Rieder et al. (2024)

### 3.2 Effect of fertilizer on soil fertility

Fertilization is one of the key factors influencing soil fertility. Different fertilization methods and application rates can have varying impacts on the physicochemical properties of the soil (Wang L. et al., 2023). Excessive application of chemical fertilizers can lead to nutrient imbalance, soil compaction, increased pH, and degradation of soil structure, thereby reducing soil quality (Hartmann and Six, 2023). Furthermore, long-term heavy use of chemical fertilizers can disrupt the soil microbial community, inhibiting the growth of certain microbial groups and affecting nutrient cycling and transformation processes (Zhang Y. et al., 2023). Studies have shown that under different fertilization treatments, the alkali-hydrolyzable nitrogen content of the soil can vary significantly, with organic fertilizer applications generally having higher nitrogen levels than chemical fertilizer treatments (Wang X. et al., 2023), indicating that organic fertilizers can significantly improve soil fertility.

Moreover, the heavy metal elements present in chemical fertilizers continuously accumulate in the soil, causing serious environmental pollution (Sun et al., 2023). The combined application of organic and chemical fertilizers can mitigate the negative impacts of chemical fertilizers to some extent, improving soil fertility and quality (He et al., 2024). The combined application of mineral and organic fertilizers does not significantly affect the pH of paddy soil, but it can significantly increase the total nitrogen, available phosphorus, and available potassium content, thereby improving soil nutrient status (Peng et al., 2023). Long-term field trials have shown that in medium to low fertility rice fields, relying solely on chemical fertilizer input is not enough to ensure stable and higher rice yield (Ma et al., 2023). The use of cow-derived organic fertilizers in South Asia and Sub-Saharan Africa has been shown to positively impact soil organic carbon, increasing it by 18–25%, as well as enhancing microbial biomass (Smith et al., 2015). It is necessary to apply organic fertilizers to improve the inherent fertility of the soil.

### 3.3 Fertilizer use and environmental impact

The application of chemical and organic fertilizers can significantly improve rice yield and soil nutrient content, but excessive use of chemical fertilizers can lead to increased greenhouse gas emissions and groundwater pollution (Wang W. et al., 2019). Studies have shown that replacing 20–40% of chemical fertilizers with organic fertilizers can reduce fertilizer usage while maintaining yield and reducing the risk of non-point source agricultural pollution (Wang R. et al., 2021). The combined application of mineral and organic fertilizers can enhance the organic matter content of soil, improve its aggregate structure, and strengthen the soil’s capacity to retain water and nutrients (Chen K. et al., 2020). These enhancements not only promote crop growth and yield but also contribute to the sustainable development of the agricultural ecosystem by improving soil health and reducing reliance on chemical inputs, which in turn mitigates environmental impacts.

The environmental benefits of applying chemical and organic fertilizers are mainly reflected in reduced greenhouse gas emissions, decreased groundwater pollution risk, and saved fossil energy consumption (Chataut et al., 2023). Compared to the use of chemical fertilizers alone, the application of organic fertilizers can significantly reduce methane and nitrous oxide emissions during rice growth (Mingcheng et al., 2024). This is mainly because the organic matter in organic fertilizers can inhibit the activity of the key enzyme - methane monooxygenase - that produces methane, thereby reducing methane generation and emission (Jiang et al., 2019). At the same time, the application of organic fertilizers can also significantly reduce the leaching of nitrates in the soil, reducing the risk of groundwater pollution (Li S. et al., 2017). This is because organic fertilizers can promote the formation of soil aggregates, improve soil’s water and nutrient holding capacity, and reduce the leaching of nutrients (Alkharabsheh et al., 2021).

From the perspective of energy consumption and carbon footprint, the production of chemical fertilizers requires a large amount of fossil energy, while organic fertilizers are mainly derived from the reuse of agricultural waste, with relatively low energy consumption in the production process. Therefore, partially replacing chemical fertilizers with organic fertilizers can to some extent reduce the consumption of fossil energy in the agricultural production process and lower the carbon emission intensity. In addition, returning straw to the field and converting livestock manure and other organic waste into organic fertilizers can realize the resource utilization of agricultural waste and reduce the environmental burden of waste disposal (Sharma et al., 2019).

## 4 Effects of fertilizer mixed application on changes of microbial community structure

### 4.1 Community diversity

The integration of mineral and organic fertilizers significantly alters the diversity of soil microbial communities. Research indicates that an increase in the proportion of organic fertilizer application correlates with a notable rise in both species richness and the diversity index of these communities (Gu et al., 2019). This phenomenon can be attributed to the high content of organic matter and nutrients in organic fertilizers, which provide a conducive substrate for the growth and reproduction of soil microorganisms, thereby promoting both microbial population growth and an enhancement in diversity (Bhunia et al., 2021). Among the various organic fertilizers, sheep manure stands out as an especially effective option due to its well-balanced nutrient composition and favorable decomposition kinetics. Table 3 presents a comparison of the nutrient profiles (N, P, K, C:N ratio) and decomposition rates of sheep manure, poultry manure, and compost, supported by recent field trial data.

**Table 3:** Nutrient composition and decomposition kinetics of sheep manure, poultry manure, and compost.

Parameter	Sheep manure	Poultry manure	Compost	References
Total N (%)	1.8–2.2	3.5–4.5	1.2–1.7	Arias et al. (2017) and Duan et al. (2021)
Total P (%)	0.5–0.8	1.2–1.8	0.3–0.6	López-Cano et al. (2016) and Li J. et al. (2024)
Total K (%)	1.0–1.5	1.5–2.0	0.5–1.0	Chen H. et al. (2020) and Tabrika et al. (2020)
C: N Ratio	15–20	10–15	20–30	Nguyen et al. (2022) and Saha et al. (2024)

Sheep manure exhibits moderate nitrogen content (1.8–2.2%) and a balanced C:N ratio (15–20), which facilitates gradual nutrient release while maintaining microbial activity (Nguyen et al., 2022). Field trials demonstrate that substituting 50% of mineral nitrogen with sheep manure (90 kg ha^−1^ N) enhances oat yield by 12–15% compared to sole mineral fertilization, attributed to improved microbial utilization of amino acids and carboxylic acids (Wang and Kuzyakov, 2024). In contrast, poultry manure, though richer in N (3.5–4.5%) and P (1.2–1.8%), has a narrower C:N ratio (10–15), leading to faster decomposition and potential nutrient leaching (Rayne and Aula, 2020). Compost, while superior in stabilizing soil organic carbon, releases nutrients more slowly due to its higher C:N ratio (20–30), making it less effective for short-term nutrient availability (Galvez et al., 2012).

The decomposition rate of sheep manure (0.012–0.018 days^−1^) strikes a balance between rapid nutrient mineralization and sustained organic matter input, fostering stable microbial diversity and enzymatic activity (Ogbete et al., 2023). Recent metagenomic analyses reveal that sheep manure application enriches *Acidobacteria* and *Firmicutes*, taxa associated with organic matter decomposition and nutrient cycling (Bhunia et al., 2021; Gao et al., 2022). These findings underscore the viability of sheep manure as a sustainable organic fertilizer, particularly in systems prioritizing long-term soil health and microbial resilience.

However, excessive application of chemical fertilizers can have an adverse effect on soil microbial diversity (Zhou et al., 2024). Large-scale application of chemical fertilizers can lead to soil acidification, inhibiting the growth of certain microbial groups and causing changes in community structure (Han et al., 2021). At the same time, the long-term use of chemical fertilizers can reduce the organic matter content of the soil, destroy the soil aggregate structure, and make the soil compact, which is unfavorable for the survival of microorganisms (Monther et al., 2020). Studies have found that under the single application of chemical fertilizers, the diversity index of soil bacteria and fungi is significantly lower than that under the mixed application of mineral and organic fertilizers (Ding et al., 2017). This indicates that the rational ratio of organic and chemical fertilizers can not only meet the nutrient needs of crops, but also maintain the diversity of soil microorganisms and promote soil health.

Considering both agricultural production and ecological benefits, the combined application of mineral and organic fertilizers is an effective way to optimize the diversity of soil microbial communities. On the one hand, chemical fertilizers can quickly supply the nitrogen, phosphorus, potassium, and other nutrients required for crop growth (Peng et al., 2023); on the other hand, organic fertilizers supplement the organic matter and trace elements lacking in chemical fertilizers, providing carbon sources and energy for microorganisms (Gondek and Mierzwa-Hersztek, 2023). The two are used in combination, ensuring a balanced supply of nutrients while promoting the improvement of microbial diversity, achieving a win-win situation for agricultural production and environmental protection. Therefore, in agricultural production practice, the application ratio of organic and chemical fertilizers should be reasonably determined according to the soil fertility status and crop needs, and the diversity of microbial communities should be optimized to improve soil quality and achieve sustainable agricultural development.

### 4.2 Microbiome changes

Soil microbial community structure is closely related to the application of mineral and organic fertilizers. Studies have found that the application of organic fertilizers can significantly increase the copy number of bacterial 16S rRNA genes in the soil, increase the diversity and abundance of microorganisms (Wang J. et al., 2024). Meanwhile, fungal ITS sequence analysis showed that the application of organic fertilizers significantly affected the composition of soil fungal communities and increased the proportion of beneficial fungi such as arbuscular mycorrhizal fungi (Liu H. et al., 2024).

These changes are mainly attributed to the input of organic fertilizers, which provide abundant carbon sources, improve soil physicochemical properties, and create favorable conditions for microbial growth and reproduction (Wu et al., 2022). Further tracking of the changes in dominant microbial species found that under long-term application of mineral and organic fertilizers, the dominant bacterial species shifted from Proteobacteria and Actinobacteria to Acidobacteria and Firmicutes (Cui et al., 2018), while the dominant fungal species shifted from Ascomycota to Basidiomycota (Ding et al., 2017). This succession in community structure reflects changes in soil nutrient status and environmental conditions. For example, the increase in the proportion of Acidobacteria bacteria, which prefer oligotrophic environments, and Firmicutes bacteria (Song et al., 2023), which can utilize complex organic matter, indicates an improvement in soil fertility. In addition to promoting the growth of beneficial microorganisms, the application of mineral and organic fertilizers can also improve the tolerance of soil microorganisms. Studies have shown that the application of organic fertilizers can enhance the resistance of microbial communities to pesticide and heavy metal stresses (Chen X. et al., 2022), which may be related to the chelation of heavy metals by organic matter and the stimulation of detoxification and resistance gene expression (Liu T. et al., 2024).

### 4.3 Changes in functional microorganisms

Soil microorganisms related to the nitrogen cycle mainly include ammonia-oxidizing bacteria, denitrifying bacteria, and nitrogen-fixing bacteria. Studies have shown that the application of mineral and organic fertilizers can significantly increase the abundance and diversity of ammonia-oxidizing bacteria in the soil, promote the nitrification of soil nitrogen, and improve the availability of soil nitrogen (Zou et al., 2022). Meanwhile, organic fertilizers are rich in organic carbon, which can provide carbon sources and electron donors for denitrifying bacteria, promote the denitrification process, and reduce the loss of nitrogen fertilizers (Hoang et al., 2022). In addition, the nitrogen-fixing bacteria in the root nodules of legumes can form a symbiotic relationship with the host plants, converting atmospheric N_2_ into amino acids that plants can absorb, replenishing soil nitrogen (Raza et al., 2020). The application of organic fertilizers can improve the living environment of rhizobia and increase the efficiency of nitrogen fixation (Lindström and Mousavi, 2020).

Phosphorus is one of the essential macronutrients for plant growth and development. It exists in the soil mainly in the form of mineral phosphorus and organic phosphorus. Microorganisms play an important role in the transformation of soil phosphorus. For example, phosphate-solubilizing bacteria can secrete organic acids to convert insoluble mineral phosphorus into soluble mineral phosphorus (Ahmad et al., 2023). Certain bacteria and fungi can secrete phosphatase to mineralize organic phosphorus into mineral phosphorus (Azeem et al., 2015). Studies have found that the application of organic fertilizers can significantly increase the number of phosphate-solubilizing bacteria and phosphatase-producing bacteria in the soil (Wang et al., 2020). This can promote the activation and transformation of soil phosphorus, and improve the utilization efficiency of phosphate fertilizers (Zhao et al., 2024).

Furthermore, there are many beneficial microorganisms in the soil that antagonize plant pathogens, such as actinomycetes that release antibiotics and pseudomonads that produce volatile antimicrobial substances (Torres-Rodriguez et al., 2022). These antagonistic microorganisms can inhibit the growth and reproduction of pathogens, reducing the occurrence of soil-borne diseases (Niu et al., 2020). The application of mineral and organic fertilizers can significantly increase the number and activity of these antagonistic microorganisms, promoting the biological control of soil-borne diseases (Sulaiman and Bello, 2024). The soil also contains a wide distribution of microorganisms that can enrich, adsorb, and transform heavy metals, playing an important role in the remediation of heavy metal pollution in farmland. For example, some bacteria and fungi can immobilize heavy metal ions through extracellular complexation, cell surface adsorption, and intracellular chelation, reducing their toxicity and bioavailability (Priya et al., 2022). The application of organic fertilizers rich in humic substances and chelating agents can provide carbon sources and energy for these microorganisms, promoting the microbial transformation and fixation of heavy metals, and reducing the risk of heavy metal contamination in agricultural products (Huang et al., 2016).

## 5 Microbial community function analysis

### 5.1 Changes of soil enzyme activity

Soil enzyme activity is an important indicator of the functional diversity of soil microbial communities, reflecting the ability of soil microorganisms to transform nutrients. Studies have shown that the activities of enzymes such as *β*-glucosidase, urease, protease, and phosphatase are significantly increased after the application of mineral and organic fertilizers (Cevheri et al., 2022). β-Glucosidase is closely related to the decomposition of soil organic matter, and increased activity can help increase the organic carbon content in the soil (Chen et al., 2016). Meanwhile, urease and protease are involved in the hydrolysis of urea and proteins in the soil, respectively, and increased activities can accelerate the mineralization rate of soil nitrogen, providing more available nitrogen sources for crop growth (Zhao et al., 2022). In addition, phosphatase plays an important role in the mineralization of organic phosphorus in the soil, and its enhanced activity can improve the availability of soil phosphorus (Li J. et al., 2021).

The application of mineral and organic fertilizers has improved the physicochemical properties of the soil, creating a favorable environment for soil microorganisms, thereby enhancing soil enzyme activity (Song Y. et al., 2022). On the one hand, the addition of organic fertilizers provides abundant carbon sources and energy materials for microorganisms, promoting the increase in microbial numbers and diversity (Han et al., 2021); on the other hand, the reasonable application of mineral fertilizers can improve the pH and nutrient status of the soil, providing suitable environmental conditions for microbial growth (Iqbal et al., 2019).

The enhancement of soil enzyme activity is of great significance for maintaining soil fertility and the stability of the agricultural ecosystem (Xiao et al., 2021). Increased enzyme activity can accelerate the cycling and transformation of nutrients in the soil, improve the availability of nutrients, and provide sufficient nutritional elements for crop growth (Sun et al., 2021). Studies have shown that there is a significant positive correlation between soil enzyme activity and crop yield, and the increase in enzyme activity can promote the absorption of nutrients and the accumulation of dry matter by crops, ultimately achieving an increase in yield and quality (Tahir et al., 2023).

Additionally, soil enzyme activity can serve as an indicator of environmental stress. When the soil is subjected to stress from heavy metals, pesticides, or other pollutants, soil enzyme activity is often inhibited (Zhang H. et al., 2020). The application of mineral and organic fertilizers can reduce the risk of agricultural pollution, improve soil environmental conditions, and enhance the tolerance of soil enzyme activity to stress (Razzaq et al., 2024). Therefore, by monitoring changes in soil enzyme activity, the health status of the soil can be timely assessed, providing a basis for agricultural pollution prevention and soil remediation (Fan et al., 2022).

### 5.2 Microbial functional gene analysis

Soil microbial functional gene analysis, based on metagenomics, can provide in-depth understanding of the metabolic potential and ecological functions of microbial communities through large-scale sequencing and functional annotation of soil microbial genomes (Rout et al., 2022). Studies have found that the application of mineral and organic fertilizers can significantly affect the abundance and distribution of soil microbial functional genes (Hu et al., 2022). For example, in paddy soils, the application of organic fertilizers can increase the abundance of functional genes related to carbon and nitrogen cycling, such as nifH and amoA genes for nitrogen fixation and ammonia oxidation (Li S. et al., 2022). This suggests that organic fertilizers can promote the nutrient transformation capacity of soil microbes (Zhang L. et al., 2023). However, excessive use of chemical fertilizers may reduce the abundance of certain functional genes, leading to changes in the metabolic functions of soil microbiota (Bai et al., 2020).

Functional annotation and metabolic pathway analysis of soil metagenomic data can comprehensively evaluate the potential of microbial communities to participate in soil element cycling and organic matter transformation (Zhao et al., 2023). Researchers have found that the long-term application of mineral and organic fertilizers significantly affects the metabolic pathways of soil microbes (Mei et al., 2021). For instance, the application of organic fertilizers can increase the abundance of genes related to carbohydrate metabolism and energy metabolism, indicating an enhancement in the metabolic activity and diversity of the microbial community (Xing et al., 2025). In contrast, the sole application of chemical fertilizers may reduce the abundance of genes in certain metabolic pathways, leading to a simplification of soil microbial functions (Huang et al., 2023).

Furthermore, the composition of soil microbial functional genes is closely related to environmental factors. Studies have shown that soil physicochemical properties, such as pH and organic matter content, significantly influence the abundance distribution of microbial functional genes (Yang et al., 2022). Therefore, the rational application of mineral and organic fertilizers, by regulating soil physicochemical properties, can optimize the structure and functions of the microbial community, thereby improving soil quality and crop yield (Shen et al., 2024).

### 5.3 Effects of microorganisms on soil fertility

The combined application of mineral and organic fertilizers directly influences the structure and function of microbial communities, thereby affecting soil fertility performance (Yang et al., 2023). Soil microorganisms are involved in the decomposition of organic matter, which is the primary process for maintaining soil organic matter balance and nutrient cycling (Coonan et al., 2020). Moderate application of organic fertilizers can increase soil organic matter content, enhance microbial activity, and accelerate the decomposition and transformation of organic matter (Rivera-Uria et al., 2024). In contrast, the application of chemical fertilizers alone often leads to microbial community imbalance, reduced organic matter decomposition capacity, and the accumulation of nutrients in the soil, which cannot be effectively utilized by crops (Cui et al., 2022). The combined application of mineral and organic fertilizers can achieve a synergistic effect, maintaining soil activity and organic matter levels, while also providing the necessary nutrients for crops in a timely manner (Ayamba et al., 2023).

In addition to influencing organic matter transformation, microorganisms also directly participate in the transformation of soil nutrients (Li Q. et al., 2023). The application of appropriate organic fertilizers is beneficial for maintaining the activity and diversity of these functional microbial groups, ensuring the effective transformation of soil nutrients (Bargaz et al., 2018). Microorganisms also play an important role in the formation of soil structure. Numerous studies have shown that certain aggregating microorganisms can secrete mucilage substances to bind soil particles into aggregates, forming a loose and porous aggregate structure (Albalasmeh and Ghezzehei, 2014). A good soil structure not only facilitates root growth but also enhances soil aeration and water permeability, preventing severe soil compaction (Shaheb et al., 2021). The application of organic fertilizers can provide a carbon source for these structural microorganisms, promoting their reproduction and improving soil structure (Li et al., 2015).

**Figure 3:**
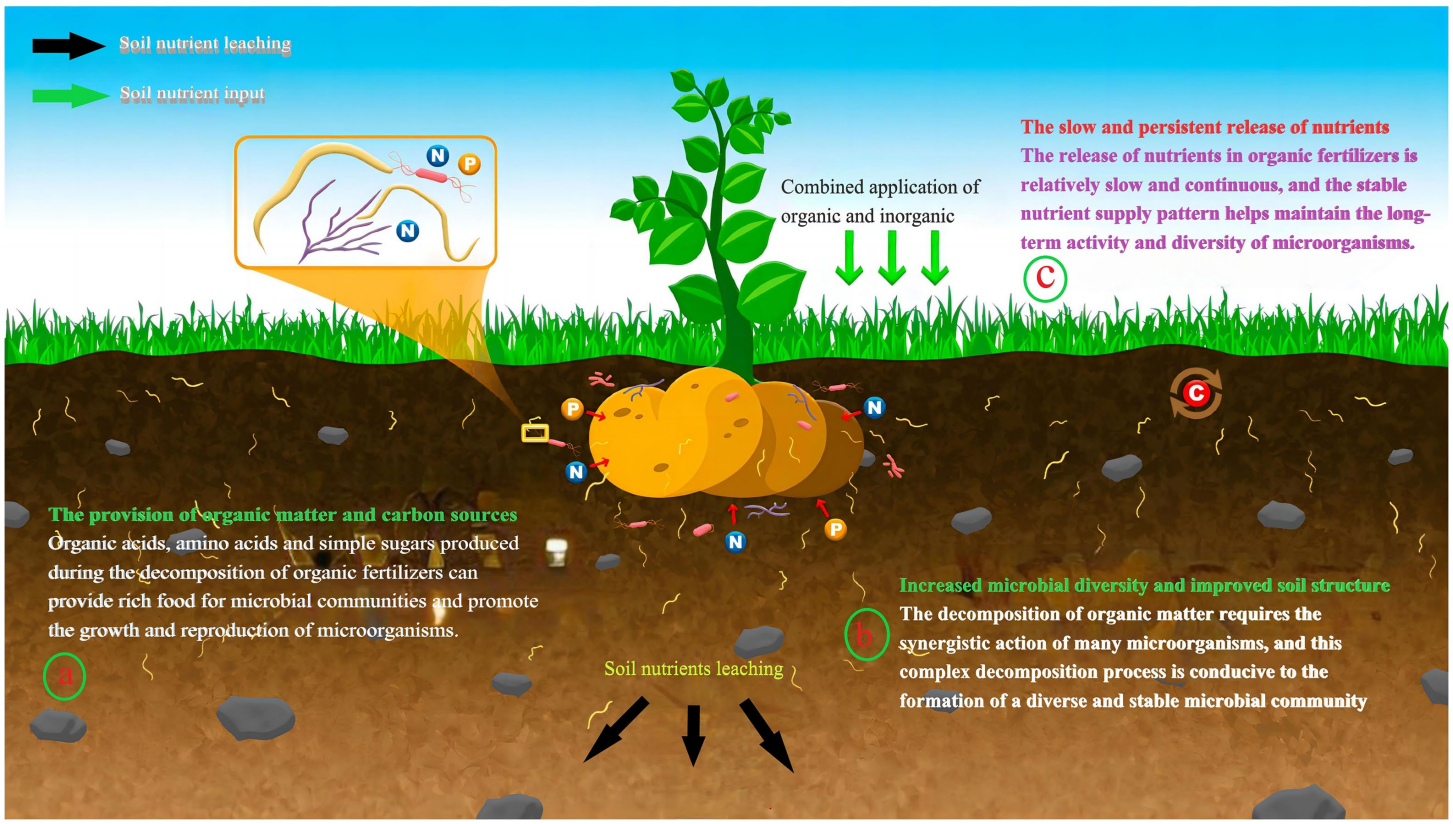
Stability analysis of mineral and organic fertilizers on microbial communities. Adapted from “Linking Nematode Communities and Soil Health under Climate Change” by Pires et al. (2023), licensed under CC BY 4.0: https://creativecommons.org/licenses/by/4.0/.

Furthermore, microorganisms can also suppress plant pathogens. Some antagonistic microorganisms can inhibit the activity of soil-borne pathogens through mechanisms such as competition for niches and the production of antibiotics, thereby reducing the occurrence of plant diseases (Niu et al., 2020). Organic fertilizers contain abundant organic matter and microorganisms, which can enhance the activity and diversity of beneficial microbial communities in the soil, improving the soil’s disease resistance (Li Q. et al., 2022). Therefore, the combined application of mineral and organic fertilizers not only directly supplements soil nutrients but also improves the structure of microbial communities, promoting the beneficial functions of microorganisms, and thereby enhancing overall soil fertility (Figure 3) (Pires et al., 2023).

## 6 Relationship between fertilizer and microbial community

### 6.1 Interaction of mineral and organic fertilizers

The long-term application of both mineral and organic fertilizers can produce synergistic effects, enhancing soil fertility and increasing crop yields (Brunetti et al., 2019). Organic fertilizers release nutrients gradually, augment the organic matter content of the soil, and improve both soil structure and the microbial environment (Tian et al., 2017). In contrast, mineral fertilizers provide nutrients rapidly, thereby supporting crop growth and development (Timsina, 2018). The combined application of these fertilizers can mitigate nutrient loss associated with chemical fertilizers, supply a carbon source and energy for soil microorganisms, and compensate for the slower nutrient release and lower nutrient content of organic fertilizers by providing crops with readily available nutrients (Dincă et al., 2022). Results indicate that the crop yield resulting from the combined application of mineral and organic fertilizers is significantly higher than that achieved with either chemical fertilizers or organic fertilizers alone (Table 4).

**Table 4:** Changes in crop yield and soil parameters due to different rates and sources of organic and inorganic fertilizers.

Fertilizer type	Crop yield increase (%)	Soil organic matter increase (%)	Available nutrient increase (%)	Microbial activity increase (%)	Reduction in nutrient leaching (%)	References
Mineral only	10–20	1–2	20–30	5–10	5–10	Timsina (2018)
Organic only	15–25	10–15	15–25	20–30	20–30	Tian et al. (2017)
Combined	25–40	15–25	30–50	30–50	30–50	Dincă et al. (2022)

The co-application of mineral and organic fertilizers can significantly influence the transformation and release of soil nutrients (Li F. et al., 2017). Specifically, the organic acids, amino acids, and other substances generated through the decomposition of organic fertilizers activate potassium, phosphorus, and other nutrients in the soil, thereby enhancing their availability (Ma et al., 2021). Additionally, humic substances form complexes with mineral nutrients, which helps reduce nutrient leaching (Chen et al., 2004). Furthermore, organic fertilizers stimulate soil microbial activity, promoting nutrient mineralization and release (Paillat et al., 2020). Research has demonstrated that the co-application of mineral and organic fertilizers increases the content of available potassium in the soil, whereas the sole application of chemical fertilizers tends to decrease available potassium levels (Choudhary et al., 2018).

The co-application of mineral and organic fertilizers significantly influences soil physicochemical properties and the micro-environment (Didawat et al., 2023). Long-term application of organic fertilizers enhances the soil organic carbon pool, improves the stability of soil aggregates, and reduces soil bulk density and compaction (Topa et al., 2021). Additionally, organic matter, including humic substances, enhances the soil’s water and fertilizer holding capacity while increasing porosity to support plant root growth (Lei et al., 2022). Furthermore, organic fertilizers provide a carbon source and energy for soil microorganisms, markedly increasing both their numbers and activity, thereby promoting soil enzyme activity and nutrient cycling (Liu et al., 2022). The application of chemical fertilizers can regulate soil pH and improve chemical properties (Li P. et al., 2021). The combined application of both types of fertilizers can optimize soil physicochemical properties and microbial community structure, creating a favorable growth environment for crops (Chen Y. et al., 2022).

The co-application of mineral and organic fertilizers offers significant benefits in mitigating the negative environmental impacts associated with chemical fertilizers (Rahman and Zhang, 2018). The sole application of chemical fertilizers can lead to soil compaction and nutrient loss, contributing to non-point source pollution (Rashmi et al., 2022). In contrast, the use of organic fertilizers can slow the migration of chemical fertilizers within the soil, thereby reducing the risk of nitrogen and phosphorus pollution in water bodies (Liu L. et al., 2021). Additionally, the co-application of organic fertilizers enhances the absorption and utilization rates of nutrients by crops, resulting in a decreased quantity of chemical fertilizers required (Zhai et al., 2022). Furthermore, some studies indicate that the combined use of organic and chemical fertilizers can diminish the uptake of heavy metals by crops, thereby improving the quality of agricultural products (Alam et al., 2020).

### 6.2 Effect of fertilizer dosage on microbial community

Soil microbial communities exhibit significant adaptability and responsiveness to varying rates of fertilizer application. Fertilizer treatments induce distinct shifts in the abundance of soil microbial groups, including Proteobacteria (34–37%), Chloroflexi (14–18%), Nitrospirae (12%), Acidobacteria (11–12%), Ascomycota fungi (41.7%), Basidiomycota fungi (27.5%), and Zygomycota fungi (25.8%) (Liu H. et al., 2021). Nitrogen fertilization initially increases microbial biomass carbon by 349% at N250 and microbial nitrogen by 250% at the same rate, along with an increase in microbial respiration of 97 and 129% at N250 and N300, respectively. However, excessive nitrogen application can result in nutrient imbalances (Babur et al., 2021). Additionally, varying fertilization rates influence the functional potential of microbial communities, including the abundance of key functional genes involved in carbon and nitrogen cycling (Chen W. et al., 2020).

The cumulative effect of fertilizer nutrients exerts a long-term influence on microbial community structure. Long-term fixed-position fertilization experiments reveal significant differences in microbial community composition under various fertilizer treatments (Chen et al., 2021). Notably, soil microbial biomass carbon content is higher in treatments that combine organic and chemical fertilizers compared to those using only chemical fertilizers or no fertilizers at all, underscoring the benefits of organic fertilizers for microbial biomass accumulation and community activity (Xu et al., 2023). Conversely, excessive use of chemical fertilizers can lead to soil acidification and inhibit the growth of specific microbial groups (Ayiti and Babalola, 2022). Furthermore, the residual effects of fertilizers can have a delayed impact on microbial communities, particularly during the initial stages of significant fertilizer regulation (Wang K. et al., 2023).

Optimal fertilizer application thresholds are essential for maintaining the stability and functional diversity of soil microbial communities. Research indicates that within these optimal fertilization ranges, indicators such as soil microbial biomass carbon, nitrogen, and enzyme activity show an increase with fertilization. However, surpassing these thresholds can lead to a suppression of microbial community structure and function (Wang Z. et al., 2021). Therefore, optimizing fertilization management and implementing balanced fertilization strategies are crucial for enhancing the beneficial roles of microbial communities in soil nutrient transformation and cycling (Li H. et al., 2024). Moreover, environmental factors, including soil type, crop species, and climatic conditions, significantly influence microbial community responses to fertilizers and must be taken into account in practical applications.

Microbial communities exhibit considerable adaptability and resilience in response to fertilizer stress. Although significant structural changes occur under long-term fertilization treatments, the high functional redundancy within these communities ensures that essential ecological processes, such as nitrogen cycling and carbon transformation, remain largely unaffected (Zhong et al., 2020). This resilience highlights the capacity of microbial communities to endure fertilizer stress (Luo et al., 2023). Furthermore, alterations in microbial community structure can serve as critical indicators for evaluating soil quality and fertility (Shi et al., 2021).

### 6.3 Stability analysis of microbial communities

Microbial community stability is essential for soil fertility and health. The combined application of mineral and organic fertilizers significantly alters the structure and function of the microbial community, resulting in increased soil carbon content and biological activity compared to the use of either fertilizer type alone (Ye et al., 2022). This mixed fertilization also enhances the levels of key soil humus components, which are crucial for maintaining microbial stability (Yu et al., 2024). The community’s resistance to stressors such as drought, high temperatures, and heavy metal pollution serves as another important measure of stability. Healthy, diverse communities can adapt their structure and function to remain stable under these stress conditions. Long-term application of organic fertilizers increases soil alkali-hydrolyzable nitrogen, while chemical phosphate fertilizers enhance phosphorus availability, thereby promoting microbial growth and stress resistance (Jiang et al., 2022). The presence of recalcitrant organic matter in fertilizers supports specific microorganisms, aiding in the maintenance of functional stability under stress.

Recovery time following disturbances is critical for assessing stability, healthy communities can rapidly restore their original structure and function. Experimental evidence indicates that mixed fertilization improves yield stability and accelerates microbial recovery compared to the use of chemical fertilizers alone (Lin et al., 2023). Key functional groups, such as actinomycetes and arbuscular mycorrhizal fungi, serve as indicators of soil health and fertility (Khaliq et al., 2022). Monitoring these groups, along with soil enzyme activities such as urease and sucrose enzymes, provides valuable insights into the metabolic activity and functional stability of the microbial community (Potts et al., 2022).

## 7 Soil sustainability assessment

### 7.1 Soil organic carbon storage

Soil organic carbon is an important carbon pool in agricultural ecosystems. The combination of mineral and organic fertilizers can improve the soil environment, increase soil organic carbon content, and increase soil carbon storage. After applying mineral and organic fertilizer, the content of complex carbon was significantly increased compared with no fertilizer, single fertilizer, and single organic fertilizer (Brar et al., 2015). The results showed that the application of mineral and organic fertilizers could improve soil colloidal activity more than that of single fertilizer and organic fertilizer (Zhang C. et al., 2022). Different fertilization measures have a significant impact on the mineralization rate of soil organic carbon and the amount of organic carbon storage (Li et al., 2018). By using the amount of organic matter returned to the field and its humification coefficient, the annual accumulated amount of soil organic carbon from crop residues and artificial fertilization can be calculated for each fertilization area (Ma et al., 2021). Based on the mineralization rate of soil organic carbon and the initial annual soil organic carbon content under different fertilization measures, the actual increase or decrease in soil organic carbon storage within 0–20 cm depth in 1 year can be calculated (Ghosh et al., 2018). In the accumulation and decomposition of soil organic matter, it is evident that the application of undecomposed corn straw is superior to that of mature organic fertilizers (Li X. G. et al., 2017).

Long-term fertilization has a lasting impact on soil organic carbon storage. It was found that the combined application of organic fertilizer and mineral fertilizer could increase the content of soil organic matter by 2.7–3.2 times (Laik et al., 2021). The optimal comprehensive effect of improving rice yield and reducing the environmental negative effect of nitrogen fertilizers is when the proportion of pure nitrogen supply in organic-mineral fertilizers is between 20 and 40%, with significant fertilizer efficiency and ecological benefits (Qiong et al., 2023).

The dynamic changes of soil carbon pools are influenced by various factors, including climate conditions, land use patterns, crop types, and agricultural management practices. In the double-season rice area, under the rice-wheat rotation system, the application of organic fertilizers and straw return is an effective way to increase soil organic carbon storage (Li D. et al., 2023). Studies have shown that straw return and organic fertilizer application can significantly increase soil organic carbon content and increase soil carbon storage (Wang et al., 2015). Meanwhile, the use of plastic mulch can increase the retention rate of organic nitrogen and reduce the loss rate, further promoting the accumulation of soil organic matter (Yang et al., 2018). The comprehensive application of various agricultural management measures to optimize the soil carbon cycle process plays an important role in enhancing the soil carbon sequestration function and mitigating greenhouse gas emissions.

### 7.2 Soil quality index evaluation

Soil quality assessment is one of the key indicators for the sustainable development of agriculture. Good soil quality can provide high-quality growth environment for crops, promote efficient nutrient utilization, and healthy development of the agricultural ecosystem (Bertola et al., 2021). Soil quality assessment needs to comprehensively consider various indicators, including soil physical and chemical properties, biological characteristics, and environmental factors (Wang et al., 2018). Among them, soil physical and chemical properties such as bulk density, porosity, pH value, cation exchange capacity, etc. are the basic indicators for evaluating soil quality, which are closely related to soil structural stability, water and fertilizer holding capacity, and nutrient availability (Maurya et al., 2020). Studies have shown that long-term application of organic fertilizers can significantly increase soil organic matter content, improve soil aggregate structure, enhance soil anti-erosion capacity and nutrient retention capacity, and play a positive role in improving soil quality (Cui et al., 2023). In addition, soil quality assessment also needs to consider soil biological characteristics, such as soil microbial biomass and enzyme activity (Paz-Ferreiro and Fu, 2016). Soil microorganisms play an important role in nutrient cycling, organic matter decomposition, and suppression of plant pathogens, which are key factors in maintaining soil health. Studies have found that long-term application of organic-mineral fertilizers can significantly increase the carbon and nitrogen content of soil microbial biomass, promote soil enzyme activities such as urease and sucrase, and accelerate soil nutrient cycling (Pu et al., 2016).

Soil fertility is another core indicator for evaluating soil quality, including soil nutrient content, nutrient availability, and fertility potential. Studies have shown that long-term application of organic fertilizers can significantly increase the total nitrogen, total phosphorus, and total potassium content in the soil, thereby improving soil fertility level (Peng et al., 2023). Meanwhile, the humic substances and amino acids in organic fertilizers can chelate metal ions in the soil, improve nutrient availability, and promote crop absorption and utilization (Zanin et al., 2019). In addition, soil fertility potential is also an important indicator for evaluating soil quality, reflecting the sustainable development capacity of soil during long-term fertilization (Vogel et al., 2019). By comprehensively considering factors such as soil nutrient content, organic matter accumulation, and aggregate structure, the soil fertility potential can be evaluated more comprehensively, providing a basis for formulating rational fertilization plans (Liu P. et al., 2023).

### 7.3 Sustainable agricultural production potential

The long-term application of mineral and organic fertilizers has a significant impact on crop yield, with soil fertility playing a key role. Studies have shown that rice yield is significantly and positively correlated with soil fertility (Wang J. L. et al., 2021). The higher the soil fertility, the more nutrients the rice absorbs from the soil, and the higher the yield (Gao et al., 2024). Meanwhile, the correlation between soil enzyme activity and crop yield is better than that between soil nutrients and crop yield (Zhang F. et al., 2023). Therefore, in agricultural production, we should not only pay attention to the use of chemical fertilizers but also focus on the use of organic fertilizers (Yu et al., 2023). Through the combined application of mineral and organic fertilizers, soil fertility can be improved, creating a good soil environment for crop growth, thereby achieving higher and more stable yields (Lu et al., 2024).

**Figure 4:**
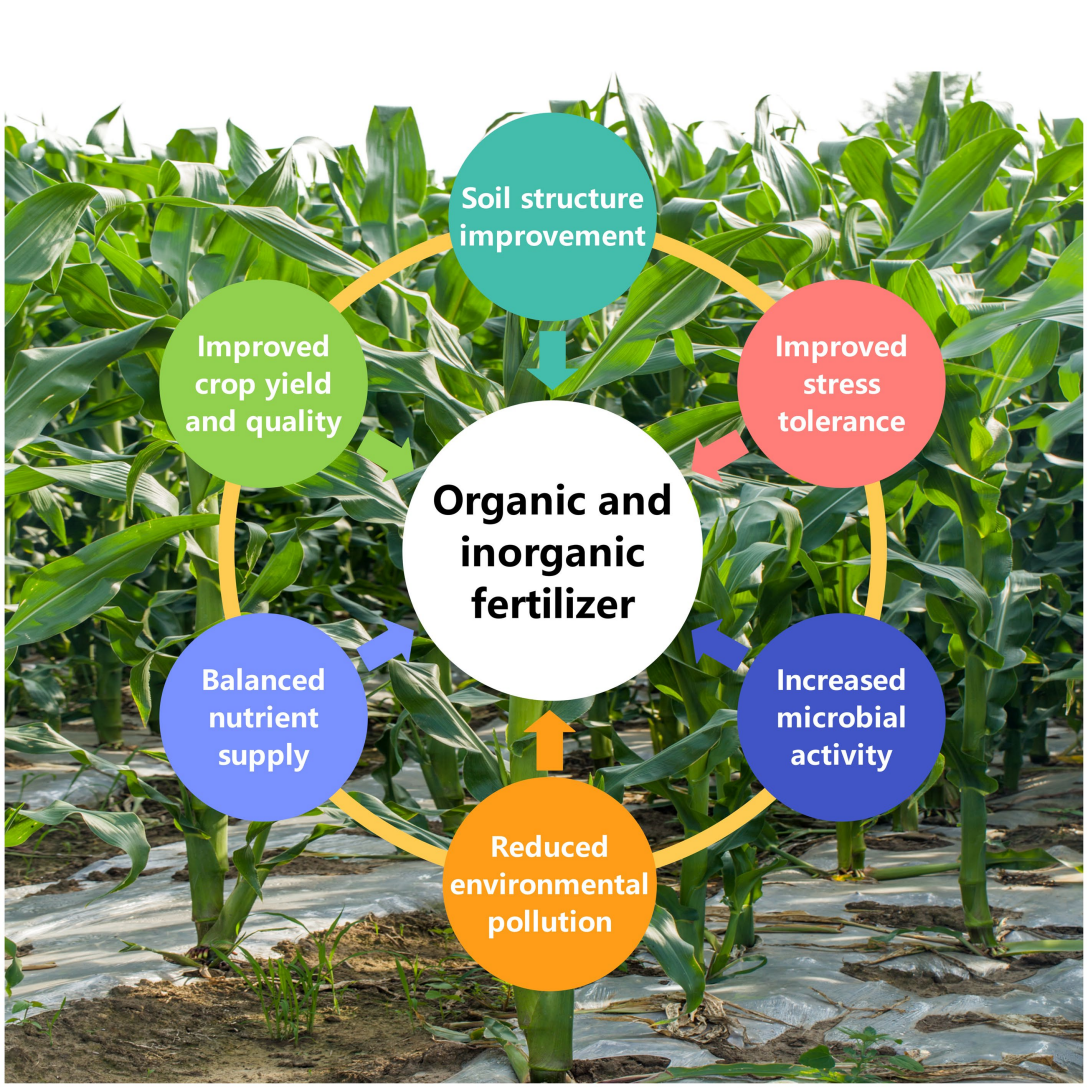
Effect of combined application of mineral and organic fertilizers on water and fertilizer utilization efficiency. Soil structure improvement, organic fertilizers contain abundant organic matter, which can improve soil structure and increase soil aggregation. This not only helps to improve soil water-holding capacity, but also enhances soil aeration. These organic matters can continuously release nutrients, providing long-term nutrition for plants, promoting root development, thereby increasing plant absorption area and nutrient absorption capacity, ultimately improving water use efficiency and crop yield. Balanced nutrient supply, mineral fertilizers can quickly provide the essential mineral nutrients such as nitrogen, phosphorus, and potassium required for plant growth, while organic fertilizers provide organic matter and some trace elements. The combined application of both can achieve a balanced nutrient supply, improve fertilizer use efficiency, and reduce nutrient loss and waste. Increased microbial activity, organic fertilizers can promote the activity of soil microorganisms. These microorganisms release plant-available nutrients during the decomposition of organic matter, and facilitate the transformation of mineral nutrients, thereby improving overall fertilizer efficiency. Improved stress tolerance, the organic matter in organic fertilizers can improve the buffering capacity of the soil, enhance plant resistance to adverse conditions such as drought and waterlogging, and thereby improve water and fertilizer use efficiency. Reduced environmental pollution, the combined application of mineral and organic fertilizers can mitigate the environmental pollution problems caused by the sole use of chemical fertilizers. The addition of organic matter helps to fix the nutrients in the soil, reducing nutrient leaching and groundwater pollution. Economic benefits, from an economic perspective, the combined application of mineral and organic fertilizers can reduce farmers’ dependence on chemical fertilizers, lower agricultural production costs, and increase economic benefits through improved crop yield and quality.

The combined application of mineral and organic fertilizers has a positive impact on the sustainability of agricultural productivity (Figure 4). The results of long-term positioning experiments show that the combined application of mineral and organic fertilizers has the highest yield sustainability coefficient (Xu et al., 2024). This is because organic fertilizers not only provide nutrients for crops but also can improve soil structure (Zhang X. et al., 2023), enhance the soil’s water and fertilizer retention capacity, and promote the activity of soil microorganisms (Chen et al., 2023), thereby facilitating nutrient cycling. Chemical fertilizers, on the other hand, mainly provide readily available nutrients to meet the growth needs of crops (Yin et al., 2018). The combined application of the two can not only meet the nutrient needs of crops but also maintain soil fertility, achieving efficient utilization of nutrients and ensuring the sustainability of agricultural production (Babu et al., 2022).

Resource use efficiency is an important indicator for evaluating the sustainability of agricultural productivity (Coluccia et al., 2020). Studies have found that under the same nitrogen input, the yield-increasing effect of applying chemical phosphate fertilizers is higher than that of chemical potassium fertilizers, and this effect is particularly pronounced in early rice (Song et al., 2024). This suggests that in the fertilization process, we should focus on the rational matching of fertilizer types to improve fertilizer use efficiency. In addition, under the combined application of mineral and organic fertilizers, the absorption of nitrogen and potassium by crops increases, and there is a surplus of nitrogen and phosphorus in the soil. This not only indicates that the combined application of mineral and organic fertilizers can improve nutrient use efficiency, but also suggests that during the application process, the fertilization amount should be reasonably adjusted according to the soil nutrient status and crop demand to avoid excessive fertilization and the resulting nutrient loss and environmental pollution.

Through the long-term combined application of mineral and organic fertilizers, it is possible to maintain or even increase yields while improving soil quality (Zhang M. et al., 2020), promoting the virtuous cycle of the agricultural ecosystem, and achieving the unity of economic and ecological benefits (Panday et al., 2024). This is of great significance for ensuring food security and promoting the green development of agriculture. In the future, long-term positioning experiments should be carried out for different regions and different crops to further reveal the yield-increasing mechanism and ecological effects of the combined application of mineral and organic fertilizers, providing a scientific basis for formulating sustainable fertilization schemes.

## 8 Environmental benefit assessment

### 8.1 Environmental risk assessment of fertilizers

The widespread application of fertilizers has not only increased agricultural productivity but also brought about a series of negative environmental impacts. Excessive application of fertilizers leads to increased greenhouse gas emissions from farmlands, such as the positive correlation between nitrous oxide emissions and nitrogen input (Menegat et al., 2022). Furthermore, heavy metals and toxic residues in fertilizers can enter surface water bodies through runoff, causing eutrophication and pollution (Craswell, 2021). Studies have shown that the nitrogen and phosphorus nutrients in fertilizers, through leaching and runoff, are one of the primary causes of surface water pollution (Liu L. et al., 2021).

The unreasonable use of fertilizers also damages the ecosystem services of the farmland. Excessive application of fertilizers can alter the physicochemical properties of the soil, destroy soil structure, reduce soil biodiversity, and thus affect soil fertility and sustainable productivity (Zhang Y. et al., 2021). Meanwhile, the production and transportation of fertilizers also consume a large amount of energy, increasing carbon emissions (Kyttä et al., 2021). Therefore, the use of fertilizers needs to consider both ensuring agricultural production and protecting the ecological environment and sustainable utilization of resources.

To reduce the environmental risks of fertilizers, it is necessary to optimize the application methods and quantities. The combined application of organic and chemical fertilizers can reduce the amount of chemical fertilizers, improve fertilizer use efficiency, and improve soil quality (Oyetunji et al., 2022). Reasonable fertilization timing and techniques also help reduce fertilizer loss and environmental impact (Duan et al., 2023). Furthermore, strengthening farmland management, such as reasonable crop rotation and straw returning, can reduce the demand for fertilizers and lower environmental risks (Xu et al., 2022). Establishing an ecological compensation mechanism for farmlands to encourage farmers to adopt environmentally friendly agricultural production methods is also an important means of controlling the environmental risks of fertilizers (Li F. et al., 2021).

### 8.2 Environmental friendliness of compost

The use of a combination of mineral and organic fertilizers can significantly improve the environmental friendliness of agricultural production. Studies have shown that when the proportion of nitrogen supply from mineral and organic fertilizers is between 10 and 30%, the overall effect on increasing rice yield and reducing the environmental impact of nitrogen fertilizers is optimal, with significant fertilizer and ecological benefits (Qiao et al., 2022). Compared to the application of chemical fertilizers alone, the combined use of mineral and organic fertilizers can increase soil organic matter content by 6.9–18.1%, improve soil physical and chemical properties, and have a negligible impact on soil pH (Zhang Y. J. et al., 2022). This indicates that the rational application of mineral and organic fertilizers can reduce the amount of chemical fertilizers used and mitigate environmental pollution risks, while ensuring crop yields (Wang X. et al., 2024).

Furthermore, the combined application of mineral and organic fertilizers can also improve nitrogen fertilizer utilization and reduce nutrient losses (Wu et al., 2020). This is partly due to the fact that plastic film mulching improves soil moisture and temperature conditions, promoting crop uptake and utilization of nutrients (El-Beltagi et al., 2022). Additionally, the combined application of mineral and organic fertilizers can promote the formation of soil aggregates, reduce nutrient leaching, and improve fertilizer use efficiency (Li et al., 2020).

From the perspective of soil fertility cultivation, the combined use of mineral and organic fertilizers has a positive impact on improving soil quality. Compared with the single application of chemical fertilizer or organic fertilizer, the combined application of organic fertilizer and mineral fertilizer significantly increased the colloidal activity and the content of soil regenerated carbon (Chen M. et al., 2022). Furthermore, long-term field trials have shown that the application of organic fertilizers alone can more effectively increase the available potassium content in the soil than the application of chemical fertilizers alone (Xin et al., 2017). These results indicate that through the scientific combination of organic and chemical fertilizers, soil aggregation can be promoted, and soil organic matter content and nutrient supply capacity can be improved, thereby enhancing soil quality (Ayuke et al., 2011).

### 8.3 Agricultural sustainable development and environmental protection

**Figure 5:**
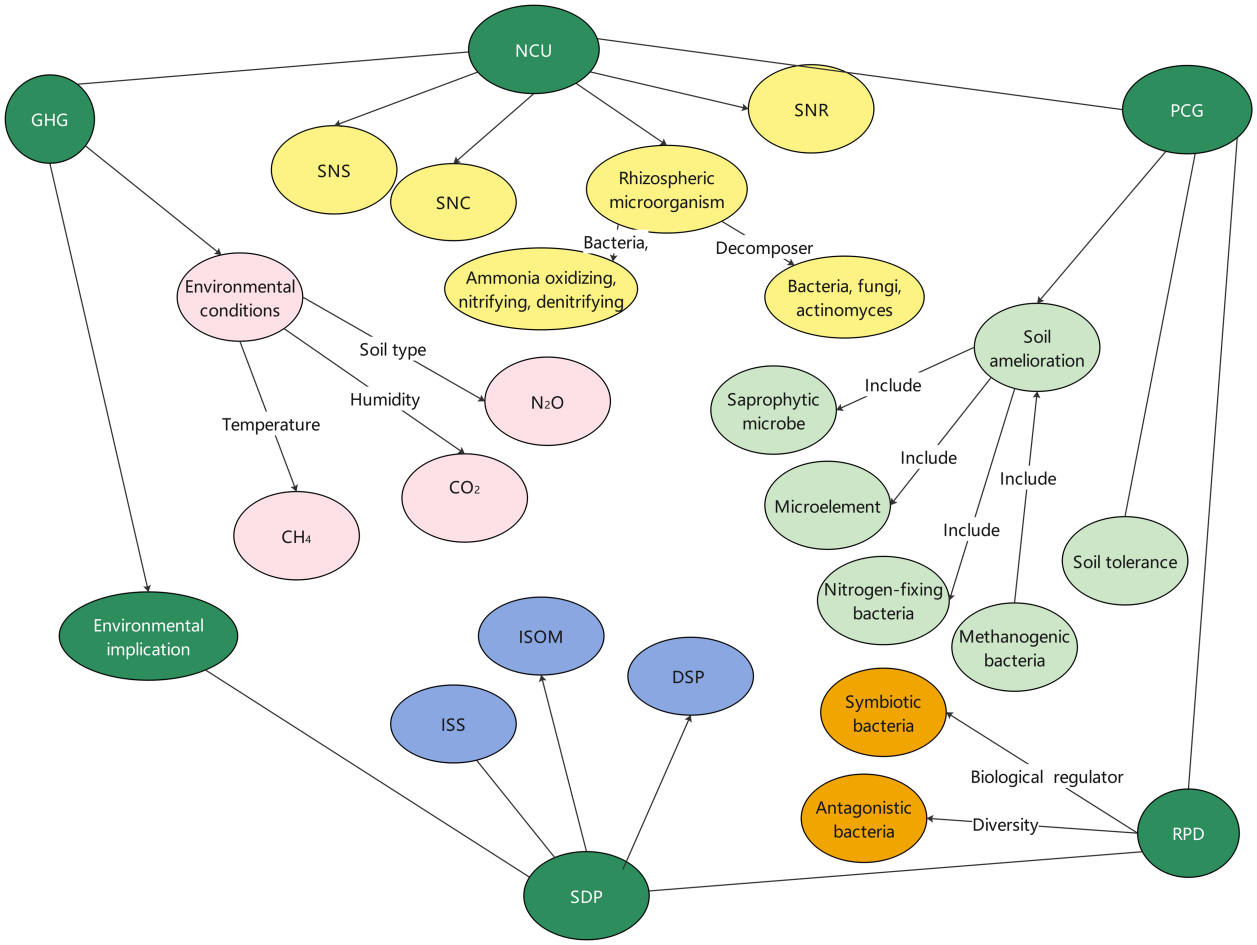
Effects of mineral and organic application on agricultural productivity and environmental benefits. GHG (greenhouse gas emission), organic fertilizers can improve soil structure and increase soil organic matter content, thereby improving soil carbon sequestration capacity and indirectly reducing greenhouse gas emissions; Environmental conditions (temperature, humidity, soil type and management) affect greenhouse gas emissions; The combined use of mineral and organic fertilizers, combined with good agricultural management practices, can improve agricultural productivity while mitigating its negative impact on climate change. NCU (nutrient cycling and utilization), it is mainly reflected in the supply, maintenance and recycling of nutrients. SNS (soil nutrient supply), organic fertilizer can provide continuous and stable nutrient supply for crops, improve soil structure, increase soil water and fertilizer retention capacity, and help plants absorb nutrients; mineral fertilizers contain a high concentration of nutrients and can quickly provide the nutrients needed for crops. SNC (soil nutrient conservation), the organic matter in organic fertilizer can increase the organic matter content of the soil, improve the soil structure, adsorb and retain nutrient elements, reduce nutrient loss, and facilitate the maintenance and utilization of nutrients. SNR (soil nutrient recycling), organic matter in organic fertilizer is decomposed by microorganisms and gradually releases nutrients; the use of organic fertilizers can promote soil microbial activity and accelerate the recycling process of nutrients. PCG (promote crop growth), the combined application of mineral and organic fertilizers can comprehensively promote crop growth and development, improve yield and quality by providing nutrients, improving soil, promoting biological activity and enhancing stress resistance. RPD (resistance to pests and diseases), organic fertilizer contains rich organic matter, improve the soil environment, help to reduce the occurrence of diseases and pests; Organic matter in organic fertilizer can promote the growth and activity of soil microorganisms, increase the number and diversity of beneficial microorganisms in soil, help plants to establish a stronger mechanism of disease and insect resistance, and improve the ability of plant disease and insect resistance; Organic matter in organic fertilizers can improve the microbial diversity of soil, and some microorganisms may inhibit the growth and reproduction of pathogens, thereby reducing the risk of the spread of diseases and pests. Soil degradation and pollution (SDP), improve soil structure (ISS), organic substances in organic fertilizers can improve the structural stability and permeability of soil, improve the physical and water retention ability of soil, and reduce soil erosion and wind erosion. Increase soil organic matter content (ISOM), organic matter in organic fertilizers can increase soil organic matter content, improve soil fertility and water and fertilizer retention capacity, promote the growth and activity of soil microorganisms, and help improve soil ecosystems. Degradation of soil pollutants (DSP), microorganisms and organic substances in organic fertilizers can promote the degradation and transformation of harmful substances in the soil, degrade pesticide residues and heavy metal pollutants in the soil, and reduce the impact of soil pollution on the environment and ecosystem.

Sustainable agricultural development and environmental protection are important goals of modern agricultural development. The technology of mineral and organic fertilizer co-application can not only increase crop yields, but also promote the formation of an agricultural circular model (Cucina et al., 2021), reduce the excessive use of chemical fertilizers and pesticides, and lower the agricultural ecological footprint (Mózner et al., 2012). The results showed that the application of mineral and organic fertilizers significantly increased the content of soil organic matter and improved soil fertility and quality (Hammad et al., 2020). By reducing the amount of chemical nitrogen fertilizer, the environmental impact of nitrogen fertilizer can be reduced while maintaining rice yield (Xue et al., 2014). Therefore, the co-application of mineral and organic fertilizers is one of the important ways to achieve sustainable agricultural development (Figure 5).

To better realize the ecological benefits of mineral and organic fertilizer co-application, it is necessary to strengthen environmental policy support. The government should introduce relevant policies to encourage farmers to adopt environmentally friendly fertilization techniques such as mineral and organic fertilizer co-application, and provide appropriate economic subsidies and technical guidance. At the same time, it is necessary to strengthen farmers’ environmental awareness education and improve their scientific fertilization ability, to avoid over-application of chemical fertilizers and cause environmental pollution. In addition, it is necessary to strengthen the monitoring and management of agricultural non-point source pollution, strictly control the discharge of agricultural production waste, and reduce pollution to soil and water bodies (Wang R. et al., 2021).

Agricultural ecosystem management is the key to achieving sustainable agricultural development. The promotion of mineral and organic fertilizer co-application technology should be combined with the optimization of agricultural ecosystem, through reasonable crop rotation, intercropping and other methods to improve land use efficiency and increase agricultural biodiversity. At the same time, strengthen the construction of agricultural water conservancy facilities, improve irrigation efficiency, and reduce water resource waste. In terms of pest control, environmentally friendly methods such as biological control should be prioritized to reduce the use of chemical pesticides. By comprehensively applying various agricultural ecological management measures, the healthy development of the agricultural ecosystem can be promoted, and the coordination of agricultural production and environmental protection can be achieved.

### 8.4 Comparative assessment of fertilizer practices across regions

The application of organic and chemical fertilizers varies significantly between developing and developed countries, influenced by socioeconomic factors, policy frameworks, and agricultural priorities. These regional differences have a substantial impact on soil health, microbial diversity, and the long-term sustainability of agricultural systems. This section synthesizes insights from the Food and Agriculture Organization (FAO) guidelines, including the International Code of Conduct for the Sustainable Use and Management of Fertilizers, along with regional case studies to elucidate key trends and challenges.

Developed nations frequently implement integrated nutrient management systems that balance the use of organic and chemical fertilizers. For example, in Japan, substituting 30–40% of chemical nitrogen with composted manure has been shown to increase microbial evenness by 20% and enhance rice yields by 10–12% (Patra et al., 2021). These practices align with FAO guidelines on balanced fertilization, which emphasize soil testing and precision agriculture to optimize nutrient application and minimize environmental impacts. European Union (EU) regulations, such as the Nitrates Directive, restrict the use of chemical fertilizers to mitigate groundwater pollution. Countries like Denmark and Germany have successfully increased microbial diversity, achieving Shannon diversity indices above 5.0, by replacing 50% of synthetic nitrogen with biogas slurry (Meegoda et al., 2025). Long-term studies in France demonstrate that combined fertilization strategies elevate the abundance of arbuscular mycorrhizal fungi by 35%, thereby enhancing phosphorus uptake efficiency (Campos et al., 2018).

In contrast, developing countries often face limited access to chemical fertilizers, resulting in a reliance on organic inputs such as manure and crop residues. While these organic practices enhance soil structure and improve microbial resilience, the lower nutrient content of organic sources frequently leads to significant yield gaps. For instance, maize yields in Ethiopia under sole organic fertilization are 30–40% lower compared to systems that integrate chemical fertilizers (Abebe et al., 2022). The FAO’s Africa Fertilizer Summit initiatives aim to address this issue by improving access to mineral fertilizers while promoting organic-integrated approaches to prevent soil degradation.

The regional disparities in fertilizer use underscore the necessity for context-specific strategies that take into account local agroecological conditions. Aligning national agricultural policies with FAO guidelines, investing in farmer education, and enhancing infrastructure are critical steps to bridging yield gaps while maintaining soil health. Future efforts should concentrate on interdisciplinary research to refine fertilization frameworks, ensuring they are adaptable to diverse agricultural environments and sustainable in the long term.

## 9 Summary and prospect

This review highlights the significant effects of combined mineral and organic fertilization practices on soil microbial communities, influencing their structure, function, and interaction networks. Such practices enhance soil microbial diversity and activity, optimize community composition, and promote the growth of beneficial microorganisms. Furthermore, mixed fertilization increases enzyme activities, particularly those involved in carbon and nitrogen cycling, such as *β*-glucosidase and urease. This indicates that combined fertilization facilitates the transformation and release of soil nutrients, thereby increasing their availability for plant uptake. These genes provide insights into microbe-driven processes related to nutrient cycling and plant growth. A comprehensive understanding of soil microbial characteristics can inform the optimization of fertilization strategies. By dynamically adjusting the ratios of organic to mineral fertilizers based on microbial diversity and community structure, it is possible to maximize ecological functions and ensure optimal crop production.
