# Correction: Enhancing soil health through balanced fertilization: a pathway to sustainable agriculture and food security

**DOI:** 10.3389/fmicb.2025.1644143

**Published:** 2025-06-24

**Authors:** Yingying Xing, Yunxia Xie, Xiukang Wang

**Affiliations:** Key Laboratory of Applied Ecology of Loess Plateau, College of Life Science, Yan'an University, Yan'an, China

**Keywords:** soil microbial community, mixed fertilizer, environmental benefits, water and fertilizer utilization efficiency, sustainable agriculture

In the published article [Pires, D., Orlando, V., Collett, R. L., Moreira, D., Costa, S. R., & Inácio, M. L. (2023). Linking Nematode Communities and Soil Health under Climate Change. Sustainability, 15(15), 11747. https://doi.org/10.3390/su151511747] was not cited in the article. The citation has now been inserted in **[5. Microbial community function analysis]**, [*5.3 Effects of microorganisms on soil fertility*], [The last paragraph] and should read:

“[Furthermore, microorganisms can also suppress plant pathogens. Some antagonistic microorganisms can inhibit the activity of soilborne pathogens through mechanisms such as competition for niches and the production of antibiotics, thereby reducing the occurrence of plant diseases (Niu et al., 2020). Organic fertilizers contain abundant organic matter and microorganisms, which can enhance the activity and diversity of beneficial microbial communities in the soil, improving the soil's disease resistance (Li Q. et al., 2022). Therefore, the combined application of mineral and organic fertilizers not only directly supplements soil nutrients but also improves the structure of microbial communities, promoting the beneficial functions of microorganisms, and thereby enhancing overall soil fertility (Figure 3) (Pires, D., et al., 2023)]”.

In the published article, there was an error in Figure 3 legend as published. The figure legend was written as “Stability analysis of mineral and organic fertilizers on microbial communities.” instead of “Stability analysis of mineral and organic fertilizers on microbial communities. Adapted from “Linking Nematode Communities and Soil Health under Climate Change” by Pires et al. (2023), licensed under CC BY 4.0: https://creativecommons.org/licenses/by/4.0/”.

The original article has been updated.

