# Correction: *Mycobacterium tuberculosis* MarR family transcription factor Rv0737 regulates bacterial growth and lipid synthesis by targeting the sigL-rslA operon

**DOI:** 10.3389/fmicb.2026.1828857

**Published:** 2026-04-07

**Authors:** Abulimiti Abudukadier, Qiao Zhang, Gang Li, Haiqi Chen, Peibo Li, Zhen Gong, Jianping Xie

**Affiliations:** 1 School of Life Sciences, Institute of Modern Biopharmaceuticals, Southwest University, Chongqing, China; 2 Chongqing Public Health Medical Center, Chongqing, China; 3 Department of Clinical Laboratory, The Second Affiliated Hospital of Anhui Medical University, Hefei, Anhui, China

**Keywords:** cell division/growth regulation, lipid metabolism, MarR transcription factor, *Mycobacterium tuberculosis*, sigL-rslA operon

In the published article the funders National Natural Science Foundation of China (No. 82211530059); Natural Science Foundation of Chongqing (No. CSTB2024NSCQ-MSX0703); Chongqing Medical Research Project (No. 2023MSXM107); Graduate Research Innovation Project of Southwest University (No. SWUB23040); and the Scientific Research Foundation of Anhui Provincial Education Department (No. 2022AH050730) were erroneously omitted.

The correct **Funding** statement is below:

“The author(s) declared that financial support was received for this work and/or its publication. This study was funded by the National Natural Science Foundation of China (No. 82211530059); Natural Science Foundation of Chongqing (No. CSTB2024NSCQ-MSX0703); Chongqing Medical Research Project (No. 2023MSXM107); Graduate Research Innovation Project of Southwest University (No. SWUB23040); and the Scientific Research Foundation of Anhui Provincial Education Department (No. 2022AH050730)”.

The original version of this article has been updated.

